# Regulation of MCP-1 chemokine transcription by p53

**DOI:** 10.1186/1476-4598-9-82

**Published:** 2010-04-20

**Authors:** Katrin Hacke, Bladimiro Rincon-Orozco, Gilles Buchwalter, Simone Y Siehler, Bohdan Wasylyk, Lisa Wiesmüller, Frank Rösl

**Affiliations:** 1 Deutsches Krebsforschungszentrum, Forschungsschwerpunkt Infektion und Krebs, Abteilung Genomveränderung und Karzinogenese, Heidelberg, Germany; 2 Universitätsfrauenklinik, Sektion Gynäkologische Onkologie, Ulm, Germany; 3 Institut de Génétique et de Biologie Moléculaire et Cellulaire, Illkirch, France; 4 Deutsches Krebsforschungszentrum, Forschungsschwerpunkt Infektion und Krebs, Abteilung Virale Transformationsmechanismen, Heidelberg, Germany; 5 Department of Medicine, David Geffen School of Medicine, University of California Los Angeles (UCLA), Los Angeles, CA, USA; 6 Dana-Faber Cancer Institute, Boston, MA 02115, USA

## Abstract

**Background:**

Our previous studies showed that the expression of the monocyte-chemoattractant protein (MCP)-1, a chemokine, which triggers the infiltration and activation of cells of the monocyte-macrophage lineage, is abrogated in human papillomavirus (HPV)-positive premalignant and malignant cells. *In silico *analysis of the MCP-1 upstream region proposed a putative p53 binding side about 2.5 kb upstream of the transcriptional start. The aim of this study is to monitor a physiological role of p53 in this process.

**Results:**

The proposed p53 binding side could be confirmed *in vitro *by electrophoretic-mobility-shift assays and *in vivo *by chromatin immunoprecipitation. Moreover, the availability of p53 is apparently important for chemokine regulation, since TNF-α can induce MCP-1 only in human keratinocytes expressing the viral oncoprotein E7, but not in HPV16 E6 positive cells, where p53 becomes degraded. A general physiological role of p53 in MCP-1 regulation was further substantiated in HPV-negative cells harboring a temperature-sensitive mutant of p53 and in Li-Fraumeni cells, carrying a germ-line mutation of p53. In both cases, non-functional p53 leads to diminished MCP-1 transcription upon TNF-α treatment. In addition, siRNA directed against p53 decreased MCP-1 transcription after TNF-α addition, directly confirming a crosstalk between p53 and MCP-1.

**Conclusion:**

These data support the concept that p53 inactivation during carcinogenesis also affects immune surveillance by interfering with chemokine expression and in turn communication with cells of the immunological compartment.

## Background

Chemokines play a crucial role in innate and adaptive immunity by attracting and activating specific subsets of effector leukocytes, cells from the monocyte/macrophage lineage as well as natural killer cells. Accordingly, these kinds of exoproteins are involved in many physiological processes such as cell proliferation, apoptosis, tumor metastasis and host defense [1]. The monocyte-chemoattractant protein-1 (MCP-1), a well-known member of the CC subgroup chemokine family, has been linked with chronic inflammatory diseases [2,3] antitumor immunity [4,5], atherosclerosis [6] and cervical cancer [7-9].

Like many other human malignancies, development of cervical cancer is a multi-step process, which is initiated by the infection of "high-risk" types of human papilloma viruses (HPV) such as HPV 16 or HPV 18. The transforming potential is encoded by the open reading frames E6 and E7. Both oncoproteins exert pleiotropic functions on their host cells, such as inactivation of the major tumour suppressors, p53 and retinoblastoma protein (pRB), respectively [10]. Although it is believed that deregulated viral oncogene expression is the first event towards anogenital cancer, the immunological defense mechanisms still have to be evaded so that progression towards cervical cancer can take place [11]. Hence, considering cancer as a micro-evolutionary process [12], "high risk" HPVs have developed several evasion or subversion strategies to escape immunological surveillance [13].

Consistent with this notion is that cervical carcinoma lines lack significant cytokine-inducible MCP-1 expression [7-9]. However, this insufficiency can be restored after fusion with normal cells, resulting in somatic cell hybrids that are no longer tumorigenic when inoculated into immunocompromised animals [14]. Notably, tumorigenic segregants derived from such hybrids again loose MCP-1 expression [9,15], indicating that abrogation of chemokine expression may provide a selective advantage for tumor progression [8]. This assumption is also supported by *in situ *hybridization and immunohistochemical studies, showing that MCP-1 expression and infiltrating cells of the monocyte/macrophage lineage were only detectable in premalignant precursor cells, but absent in high-grade lesions of cervical cancer patients [7,16,17].

Concerning MCP-1 expression, previous studies have identified both the nuclear factor-kappa B (NFκB) and the activator protein-1 (AP-1) as essential regulators of MCP-1 transcription and its induction upon TNFα stimulation [18-21]. TNF-α is a multifunctional cytokine that is involved in many regulatory circuits such as cell cycle control, interferon-β induction, inflammation and apoptosis [22-24]. Binding of TNF-α to its cognate receptors (TNF-R1/-R2) particularly results in the activation of AP-1 and NFκB [24,25]. On the other hand, TNF-α can also induce p53 which modulates a variety of genes involved in apoptosis, DNA repair and innate immunity [26-29]. TNF-α activates p53 via NFκB by binding to the corresponding promoter [30], which is, however, counteracted by the HPV-E6-mediated degradation of p53 through the proteasomal pathway [31] or by p53 mutations in virus-independent forms of human cancer [32].

In the present study, we demonstrate that p53 is directly involved in the regulation of human MCP-1 transcription. This supports the notion that p53 inactivation and consequently MCP-1 down-regulation during HPV-induced pathogenesis generates a selection advantage during progression to cervical cancer, explaining the often observed successive depletion of immunological effector cells within late premalignant HPV-positive lesions.

## Methods

### Cell lines and cytokine treatment

Primary human foreskin keratinocytes (HFKs) were immortalized with E6, E7 or E6/E7 oncogenes of HPV16 [33] and maintained in Keratinocyte Growth Medium (PromoCell, Heidelberg, Germany). Early and late passage MDAH041 Li-Fraumeni cell lines [34-36], non-tumorigenic somatic cell hybrids ("444") between HeLa and IMR-90, their tumorigenic segregants ("CGL3") [14] and the human glioblastoma cell line A172 were cultured as previously described [22]. Stable Hep3B clones [37] carrying puromycin resistance alone ('BT2E') or in combination with stable expression of the temperature sensitive mutant p53val135 ('4Bv') were cultivated in Dulbecco's modified Eagle's medium, supplemented with 10% fetal calf serum, 1% penicillin/streptomycin and additionally 2 μg/ml puromycin (Sigma) except during experiments. For cytokine treatment, the cells were incubated with 250 U/ml TNF-α (Strathmann Biotec AG) for different periods of time as indicated in the figure legends.

### Transfection

A172 cells (1 × 10^5^) were transfected with Effectene Transfection Reagent (Qiagen) according to the manufacturer's instructions using 1 μg pSUPER-p53 for the formation of siRNA targeting p53 transcripts or the respective empty vector [38] as described in the figure legend.

### Lentiviral shRNA-mediated knockdown of p53 in HPV E7 immortalized keratinoytes

Cells were infected either with Lentiviruses encoding short hairpin shRNA targeting p53 (Santa Cruz Biotechnology, sc-29435-v) or scrambled controls (sc-108080) according to the manufacturer's protocol. Briefly, infection was carried out at a multiplicity of infection (MOI) of 5 in serum free growth medium containing 5 μg/ml of polybrene at 37°C and 5% CO_2_. After 4 hours, serum contained medium was added and the cells were cultured for 48 hours. After 2 day post-infection, infected cells were selected in the presence of puromycin (5 μg/ml) for 5 days. RT-PCR analyses for p53 knockdown efficiency and MCP-1 transcription was performed as described below.

### SDS-PAGE and Western blotting

Whole cellular proteins were separated in 10-12% sodium dodecyl sulfate (SDS) polyacrylamide gels (PAGE) and electrophoresed to PVDF membranes (Immobilon-P, Millipore Corporation). The following antibodies were used: p53 (DO-1) from Santa Cruz, phospho-Thr^180^/Tyr^182 ^p38 MAPK (9211S) and p38 MAPK (9212) from NEB. Equal protein transfer and loading was routinely controlled by incubating the filters with a monoclonal actin antibody (ICN Biomedicals).

### Northern blotting

Total cellular RNA was isolated with the RNeasy kit (Qiagen) according to the instructions of the manufacturer. 4-6 μg of RNA were separated on 1% agarose gels in the presence of ethidium bromide under nondenaturing conditions and transferred to GeneScreen Plus membranes (DuPont, NEN). Specific probes for the hybridization were labeled with [^32^P]dCTP (Amersham) by random priming using HexaLabel Plus DNA Labeling Kit (Fermentas). The filters were hybridized in 50% formamide, 10 U/ml tRNA, 5 × SSC, 0.1% Denhardt's-buffer, 50 mM sodium phosphate-buffer, 1% SDS. Washing was performed in 2 × SSC with 0.1% SDS at 68°C. Hybridized filters were exposed to high performance autoradiography films. Densitometric quantification of band intensities was performed using a ChemImager 5500 with software (Alpha Innotech Corporation, San Leandro, California, USA), analyzing signals within the linear range.

### DNA hybridization probes

The full-length cDNA encoding the monocyte-chemoattractant-protein-1 (MCP-1) [39] was obtained from the American Type Culture Collection (Rockville, MD, USA). The cDNA for p21 [40] was kindly provided by B. Vogelstein (John Hopkins Institute, Baltimore, MD) via P. Jansen-Dürr (University of Innsbruck, Austria). The plasmid pc-myc containing the third exon of the human c-myc gene was kindly made available by G. Bornkamm (Institut für Klinische Molekularbiologie, München, Germany) [41]. The cDNA of human β-actin was a generous gift from H. Bierhoff (DKFZ, Heidelberg).

### Semiquantitative RT-PCR

Total cellular RNA was isolated using the RNeasy Kit (Qiagen, Turnberry Lane, CA). For RT, 1 μg of DNase treated RNA was prepared as described previously [22]. PCR was performed using Taq Polymerase (Promega) according to the manufacturer's instructions using the following primers: MCP-1 sense 5'-CAG ATG CAA TCA ATG CCC CAG T-3'; MCP-1 antisense 5'-ATA AAA CAG GGT GTC TGG GGA AAG C-3', IFN-β sense 5'-GAT TCA TAT AGC ACT GGC TTG-3', IFN-β antisense 5'-CTT CAG GTA ATG CAG AAT CC-3'. p53 sense 5'-AGA GCC ACC GTC CAG GGA GC-3' antisense 5'-GGC AGT GAC CCG GAA GGC AG-3'. Conditions for p53, MCP-1 and IFN-β PCR: 3 min at 94°C, followed by 30 cycles (35 cycles were applied for MCP-1) for 30 s at 94°C, 1 min at 58°C, 30 s at 72°C and 10 min at 72°C. GAPDH primers: sense 5'-TGG ATA TTG TTG CCA TCA ATG ACC-3'; antisense 5'-GAT GGC ATG GAC TGT GGT CAT G-3'; amplification was made as previously described using 65°C of annealing temperature. The PCR products were analyzed on agarose gels.

### Cell cycle analysis

Cells were harvested by trypsination, washed twice with phosphate-buffered saline (PBS) and fixed overnight with 70% ethanol. After fixation and centrifugation, the cell pellets were resuspended in DNA staining solution containing DAPI (5 × 10^-6 ^M) as the DNA dye and SR 101 (5 × 10^-6^M) as a protein counter stain as described by Stöhr et al. [42]. Processing, cell cycle analysis of flow cytometric data were performed according to Dean and Jett [43].

### Electrophoretic mobility shift assays with p53 isolated from insect cells

p53 proteins from human origin were expressed in High Five™ insect cells and isolated as described previously [44,45]. DNA binding experiments were performed in a reaction mixture containing 60 ng of p53 protein, 2 nM of the labeled probe and 20 nM of competitor tRNA in 25 mM Tris-HCl (pH 8.0), 5 mM EDTA, 1 mM DTT and 6% glycerol. In control reactions without p53, the protein storage buffer was added. For supershift analysis the mixtures were preincubated with the indicated antibodies for 30 min before addition of labeled DNA. Samples were loaded onto a 4% native polyacrylamide gel and electrophoresed in 6.7 mM Tris-HCl (pH 8.0), 3.3 mM sodium acetate and 2 mM EDTA. After electrophoresis gels were dried and exposed to X-ray films. To obtain radioactive DNA substrates, oligonucleotide #1 was end-labeled with T4 polynucleotide kinase and [γ-^32^P] ATP and annealed with unlabeled, complementary oligonucleotide #2. Double-stranded DNA molecules were purified in 8% native polyacrylamide gels. DNA concentrations were measured by using DNA Dipsticks (Invitrogen). The following oligonucleotides covering the wild-type p53-specific RGC, RGC mutated and the MCP-1 p53-site were used: RGC-#1: 5'-TCG AGT TGC CTG GAC TTG CCT GGC CTT GCC TTT TC-3', RGC-#2: 5'-GAA AAG GCA AGG CCA GGC AAG TCC AGG CAA CTC GA-3', mutRGC-#1: 5'-TCG AGT TTA ATG GAC TTT AAT GGC CTT TAA TTT TC-3', mutRGC-#2: 5'-GAA AAT TAA AGG CCA TTA AAG TCC ATT AAA CTC GA-3', MCP-#1: 5'-AGA GAT GAC AAC TCC TTC CTG AAG TAG AGA CAT GCT TCC AA-3', MCP-#2: 5'-TTG GAA GCA TGT CTC TAC TTC AGG AAG GAG TTG TCA TCT CT-3'. Monoclonal antibodies used for p53 supershift analyses were: DO-1 (Ab-6), PAb1801 (Ab-2), and PAb421 (Ab-1) directed against the amino acids 21-25, 32-79, and 372-382, respectively (Calbiochem); ab9484 directed against GAPDH from Abcam.

### Chromatin Immunoprecipitation

Experiments were performed with the chromatin immunoprecipitation (ChIP) assay kit (Upstate Biotechnology) according to the manufacturer's instructions except that the LiCl Immune Complex washing buffer was diluted 1:5 in H_2_O before use. 1 × 10^6 ^cells were used per immunoprecipitation reaction mixture. Cells were crosslinked in 1% formaldehyde for 10 min, washed twice in PBS and lysed in SDS lysis buffer (50 mM Tris-HCl, pH 8.1, 10 mM EDTA, and 1% SDS) and Complete protease inhibitor cocktail (Roche) on ice for 10 min. The chromatin was sonicated to an average length of 500-1000 bp. The sonicated cell supernatant was 10 fold diluted in ChIP dilution buffer (16.7 mM Tris-HCl, pH 8.1, 1.2 mM EDTA, 0.01% SDS, 1.1% Triton X-100 and Complete protease inhibitor cocktail, Roche) and precleared by incubating with salmon sperm DNA/protein A agarose-beads for 1 h at 4°C. An aliquot of the diluted cell supernatant (5%) was kept to quantitate the amount of DNA present in different samples (input). The protein A agarose beads were then pelleted and discarded, and the protein-chromatin complexes were immunoprecipitated with an antibody against p53 (8 μg, FL-393; Santa Cruz) or rabbit immunoglobulin G (8 μg, IgG; Santa Cruz) at 4°C over night. Each reaction mixture was then incubated with salmon sperm DNA/protein A agarose beads for 1 h at 4°C. The protein A agarose beads/antibody/chromatin complexes were washed once with Low salt immune complex wash buffer (20 mM Tris-HCl, pH 8.1, 150 mM NaCl, 0.1% SDS, 1% Triton x-100 and 2 mM EDTA), once with High salt immune complex wash buffer (20 mM tris-HCl, pH 8.1, 500 mM NaCl, 0.1% SDS, 1% Triton X-100 and 2 mM EDTA), once in LiCl immune complex wash buffer (10 mM Tris-HCl, pH 8.1, 1 mM EDTA, 0.25 M LiCl, 1% IGEPAL-CA630 and 1% deoxycholic acid,) and twice in TE-buffer (10 mM Tris-HCl, pH 8.0, 1 mM EDTA). The protein-chromatin complexes were eluted twice from the protein A agarose beads by use of IP elution buffer (0.1 M NaHCO_3_,1% SDS), followed by reverse cross-linking in 0.3 M NaCl at 65°C for 4 h. Each reaction mixture was then incubated with 40 mM Tris-HCl, pH 7.4, 0.2 M NaCl and 2 μl of 10 mg/ml Proteinase K at 45°C for 1 h. Subsequently, the samples were purified by phenol-chloroform extraction. PCR analysis of the isolated DNA fragments used the following primer pairs: MCP-1 (295 bp fragment primers) forward, 5'-GCAGAAGTGGGAGGCAGACA-3', and reverse 5'-GGCTTATAGACA TCCTGTGGCATG-3', MCP-1 (nested primers) forward 5'-TTCTGATTCATACCCTTCACC TTCC-3' and reverse 5'-GATAAAGCCACAATCCAGAGAAGGA-3' p21 forward, 5'-CTGTGGCTC TGATTGGCTTT-3', and reverse 5'-ACACAAGCACACATGCATCA-3'. PCR reactions with the different primers gave rise to a single specific product of the expected size. PCR products were resolved on 2% agarose gels.

## Results

### MCP-1 expression in HPV16 E7 immortalized keratinocytes and detection of a p53 binding site within the enhancer region

**Figure 1:**
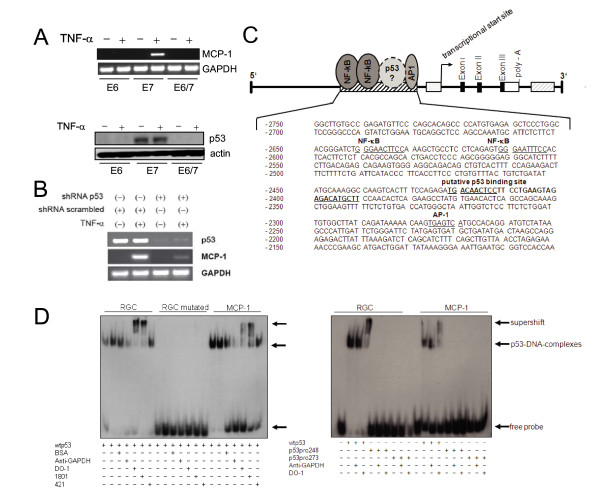
**MCP-1 expression in HPV16 immortalized human keratinocytes and in vitro binding activity of wild-type p53 at the MCP-1 regulatory region**. **(A) Upper panel**: RT-PCR for MCP-1 and GAPDH in HPV16 E6-, E7-, and E6/7 immortalized human foreskin keratinocytes. Cells were treated with TNF-α (250 U/ml) (+) for 6 h. (-) untreated control. The RT-PCR products were separated on 1.5% agarose gel. **Lower panel**: Western blot analysis of p53. Cytosolic extracts (50 μg per lane) were separated in a 12% SDS-PAGE. Equal loading was confirmed using an actin specific antibody. (**B**) RT-PCR for MCP-1, p53 and GAPDH in E7-immortalized keratinocytes after lentiviral p53 shRNA delivery. Cells were treated as described in panel A. **(C) **Schematic overview of the MCP-1 gene. The region -2750 to -2150 bp upstream of the MCP-1 start site harbors two NFκB and one AP-1 binding site. The *in silico *identified putative p53 binding site (-2422/-2391) relative to NFκB and AP-1 is indicated. Black boxes: exon I-III; white box: polyadenylation site (poly-A), (striped grey box): 3'-regulatory region. **(D) Left panel**: Electrophoretic mobility shift assay (EMSA): 60 ng of recombinant p53 protein was incubated with [^32^P]-labeled double-stranded oligonucleotides harboring the wild-type p53-specific RGC-site, the mutated version thereof and the putative MCP-1 p53 binding site, respectively. In addition, binding was also performed in the presence of 200 ng of BSA, anti-GAPDH, DO-1, PAb1801, or PAb421. The positions of free probe, DNA in complex with p53, and DNA in complex with p53 plus supershifting antibodies are indicated by arrows. **(D) Right panel**: EMSA: comparative binding analysis for wild-type p53 (wtp53) using specific RGC and non-specific RGC mutated versus MCP-1 DNA. DNA binding activities of wild-type p53 (wtp53), p53pro248 and p53pro273 mutants are indicated.

Previously we have shown that in contrast to primary human keratinocytes, MCP-1 expression and its inducibility by TNF-α is lost during cellular immortalization by HPV16 [8]. Conversely, using amphotropic retroviruses that separately encode the oncoproteins of HPV16 (E6, E7 and E6/E7, respectively), TNF-α is only inducing MCP-1 in E7-positive keratinocytes, but not in cells expressing either E6 alone or in conjunction with E7 (Fig. 1A). Since this correlates with the presence of p53 [33], we monitored MCP-1 inducibility in E7-immortalized keratinocytes after delivery of lentiviral vectors encoding a specific short hairpin shRNA directed against p53. For this purpose, cells were infected in parallel with a scrambled control and selected in the presence of a dominant selection marker. As shown in Fig. 1B, knock-down of p53 has a strong negative impact on MCP-1 induction after TNF-α treatment, suggesting an involvement of p53 in MCP-1 gene regulation. Indeed, monitoring the upstream region by *in silico *analysis, a potential p53 binding site within the MCP-1 promoter was predicted.

Fig. 1C schematically depicts the 5'-region of MCP-1 2.7-2.2 kb upstream from the transcriptional start site, which is characterized by the presence of a prominent DNAseI hypersensitive site (DHSS) when the nucleosomal organization of the MCP-1 gene is examined [18]. By scanning the 5'-DHSS for additional DNA binding motifs, a putative p53 binding sequence (5'-tGACAAcTCCxxxxxxxxxxxxAGACATGCTT-3'), located between AP-1 and NFκB binding sites, could be detected. This sequence shows high homology (18 out of 20 bp) with the p53 consensus site (5'-(PuPuPuC(A/T)(T/A)GPyPyPypY)_n_-3') and comprises two half-sites separated by a spacer of 12 bp, which is within the normal range between 0-13 bp [46].

To determine whether p53 can physically interact with this sequence, electrophoretic mobility shift assays (EMSAs) using a 41-mer double-stranded oligonucleotide encompassing the putative binding site in MCP-1 were performed. Human wild-type p53 protein was obtained from baculovirus infected insect cells and p53-DNA complex formation was verified using the sequence-specific p53 recognition site from the ribosomal gene cluster repeat (RGC) as positive and a mutated version as negative control [47]. As shown in Fig. 1D, both MCP-1 and RGC oligonucleotides form distinct bands with the same mobility, indicating that similar p53-DNA complexes were formed, while mutated RGC failed to bind (left panel). To demonstrate the specificity of wild type p53 in DNA-complex formation, supershift analyses with monoclonal anti-p53 antibodies (DO-1, PAb1801, and PAb421, respectively) were carried out. Addition of DO-1 and PAb1801 antibodies caused retardation of p53-DNA complexes, while BSA and purified monoclonal antibody against GAPDH as controls did not. PAb421 slightly enhanced p53-DNA complex formation but did not induce a supershift. This difference may be due to the phosphorylation pattern of baculovirus derived p53, since the PAb421 epitope only becomes exposed when the Ser376 residue is dephosphorylated [48]. To further ascertain the specificity, we analyzed the affinity of a DNA-contact mutant (p53pro248) and a conformational mutant of p53 (p53pro273) [49], which both failed to produce a gel shift (Fig. 1D, right panel). On the basis of the *in vitro *binding studies, one can conclude that wild-type p53 forms stable complexes with the *in silico *predicted p53 binding site within the MCP-1 upstream regulatory sequence.

### Role of p53 in TNF-α-mediated MCP-1 induction

To further delineate the function of p53 in the regulation of the MCP-1 gene, we used the p53 negative hepatocellular carcinoma cell line Hep3B, stably co-transfected with an expression plasmid encoding a murine temperature-sensitive mutant of p53 (p53val135) and a vector for puromycin resistance ("4Bv") or with the resistance marker alone ("BT2E"). Shifting the temperature of 4Bv cell cultures to 32°C restores wild-type properties of p53 whereas at 37°C p53 is inactive [37].

**Figure 2:**
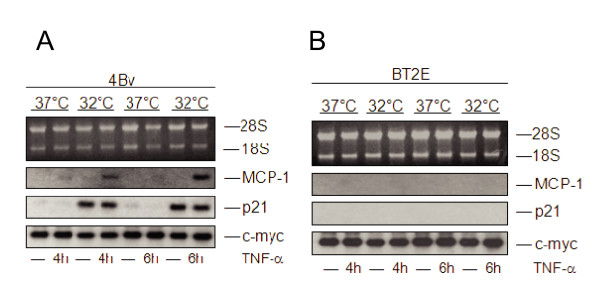
**MCP-1 induction by TNF-α after reconstitution of wild-type p53 activities**. 4Bv **(A) **and BT2E **(B) **cells were cultivated at 37°C or shifted to 32°C overnight. Stimulation was done with TNF-α for 4 h or 6 h. Total cellular RNA was separated in a 1% agarose gel and transferred to a Gene screen Plus membrane. The filter was subsequently hybridized with a p21, MCP-1 and c-myc cDNA probe. The positions of the 28S and 18S ribosomal RNA are indicated.

As depicted in Fig. 2A, transcription of p21 mRNA was strongly induced in 4Bv cells after the shift to 32°C, when compared to the cells left at 37°C, independently of whether TNF-α was added or not. In BT2E control cells, however, no p21 induction was observed (Fig. 2B). This clearly indicates that p53 in 4Bv cells was functionally reconstituted at 32°C.

In order to test whether p53 in 4Bv cells affects MCP-1 gene regulation, the same filters were hybridized with a MCP-1 cDNA probe. As shown in Fig. 2A, induction of MCP-1 after stimulation with TNF-α was detectable in 4Bv cells, but only at 32°C. Induction was selective, since the immediate-early gene c-myc (Fig. 2A) was not modulated under these conditions. In contrast, induction of MCP-1 was not detected in BT2E cells after temperature shift and stimulation with TNF-α (Fig. 2B). This indicates that reconstitution of p53 activity restores the inducibility of MCP-1 transcription by TNF-α.

### Cell cycle distribution and TNF-α signaling are not affected in 4Bv cells by temperature shift

**Figure 3:**
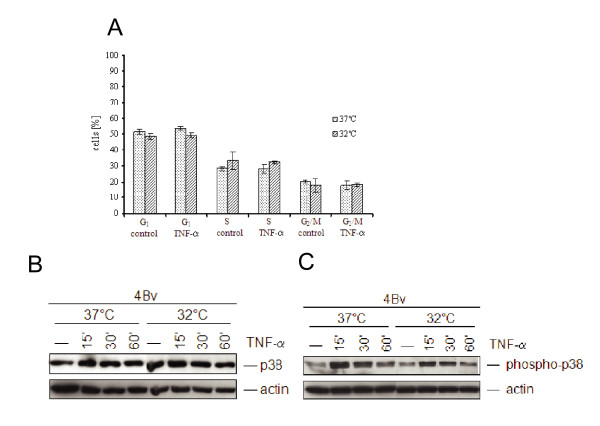
**Temperature shifting does not affect the cell cycle or TNF-α signaling**. **(A) **4Bv cells were maintained at 37°C or 32°C overnight, followed by treatment with or without TNF-α for 6 h. Flow-cytometric analysis was performed on DAPI/SR 101 stained cells to determine the percentage of 4Bv cells in different cell cycle phases (G1, S, G2/M). Mean values (columns) and standard deviations (bars) are given for three independent experiments. Steady state expression **(B) **and phosphorylation **(C) **of p38 MAP kinase was analyzed after treatment of 4Bv cells (at 37°C or 32°C) with TNF-α as indicated. Total cellular protein (50 μg) was separated in a 12% SDS-PAGE gel. After electrotransfer, the filter was consecutively incubated with antibodies as indicated and re-probed with anti-actin antibody as loading-controls. (-): untreated control cells; (15')/(30')/(60'): cells treated with 250 U/ml of TNF-α for the indicated time.

Although activation of threshold levels of wild-type p53 and p21 does not induce growth arrest in 4Bv cells [37], we wanted to exclude the possibility that increased MCP-1 expression may simply result from a kind of synchronization effect, accumulating more cells in cell cycle phases that can re-express MCP-1 upon TNF-α addition than a non-synchronized cell population. However, flow cytometric analyses of cells growing at different temperatures revealed no significant changes in their percentage distributed in different phases of the cell cycle, indicating that synchronization cannot account for the stronger MCP-1 expression (Fig. 3A).

Moreover, to rule out that temperature-shifts may affect TNF-α signaling, activation of p38 MAP kinase was examined [50]. For this purpose, cells were cultivated at 32°C or 37°C, treated with TNF-α for different periods of time and analyzed for p38 phosphorylation via immunoblotting. As presented in Fig. 3, there were equal amounts of non-phosphorylated p38 (panel B), which became phosphorylated with roughly the same kinetics after TNFα addition, independently of the temperature shift (panel C).

### Induction of MCP-1 by TNF-α depends on p53 expression

**Figure 4:**
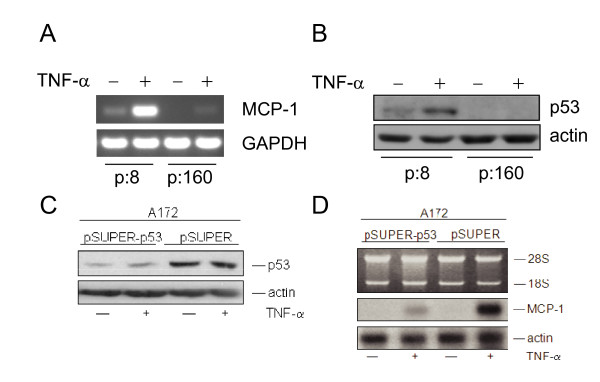
**Expression of MCP-1 in Li Fraumeni fibroblasts and after *p53 *knockdown in A172 cells**. **(A) **RT-PCR of MCP-1 and GAPDH in Li Fraumeni cells (MDAH041) in passage 8 (p:8, p53 mut/wt) and passage 160 (p:160, p53 mut/mut), respectively. Cells were treated with 250 U/ml of TNF-α for 6 h (+). Untreated control cells: (-). **(B) **Western blot analysis of p53 in MDAH041 cells (p:8) and (p:160) treated with TNF-α (250 U/ml) (+) for 6 h. (-): untreated control. Cytosolic extracts (50 μg per lane) were separated in a 12% SDS-PAGE. Actin confirms equal loading. **(C) **A172 cells were transiently transfected with pSUPER-p53 or with the empty pSUPER vector. After 24 h, cells were stimulated with TNF-α for additional 5 h. Cells were harvested and Western blot analysis was performed. Filters were probed with anti-p53 (DO-1) (see also panel B). **(D) **4 μg of total RNA were separated in a 1% agarose gel and transferred to a Gene screen Plus membrane. The filter was subsequently hybridized with a MCP-1 cDNA probe. Hybridization of the same filter with a cDNA probe coding for the housekeeping gene β-actin confirmed equal loading. The positions of the 28S and 18S ribosomal RNA are indicated.

To further prove a more general role of p53 in MCP-1 regulation, early passage immortalized cells from a Li-Fraumeni patient were used. These fibroblasts initially carry a germ-line mutation in one p53 allele [51]. After longer *in vitro *cultivation, however, they spontaneously immortalize at a high rate, with frequent somatic mutations in the remaining wild-type allele of p53 [34,35]. As demonstrated in Fig. 4A, cell cultures at passage 8 still showed a significant MCP-1 induction after TNFα treatment, which was strongly reduced when immortalized cells at passage 160 were examined. The usage of the p53 specific DO-1 antibody illustrates that wt-p53 can still be detected and induced by TNF-α in early, but not in late passage cells (Fig. 4B), carrying a frameshift mutation at amino acid position 175 (changing arginine to histidine) [36,52,53].

Moreover, to prove the impact of endogenous p53 knockdown also in other cell systems, we used the glioblastoma cell line A172 (Fig. 4D), where similar to E7-immortalized keratinocytes (see Fig. 1A, upper panel), expression of MCP-1 can be strongly induced by TNF-α [20]. After transient transfection with pSUPER-p53 which encodes a p53 specific siRNA [38] and subsequent stimulation with TNF-α, suppression of p53 (Fig. 4C) and a reduced MCP-1 transcription could be also discerned (Fig. 4D). Taken together, these data prove the notion of a wide-ranging involvement of p53 in MCP-1 inducibility in different cell types.

### p53 binding to the MCP-1 5'-regulatory region in vivo

**Figure 5:**
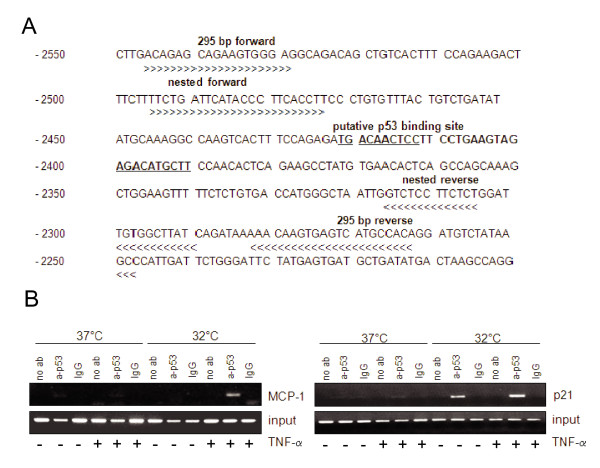
**p53 binds to the enhancer region of *MCP-1 in vivo***. **(A) **DNA sequence of the 5'-regulatory region of the MCP-1 gene (-2550 to -2250) indicating MCP-1 specific primer pairs used for the chromatin immunoprecipitation (ChIP) assay by arrows. The p53 binding site is marked with bold letters. **(B) **4Bv cells were maintained at 37°C or shifted to 32°C for 5 h and stimulated with TNF-α for additional 5 h. Cells were harvested for chromatin immunoprecipitation (ChIP) assay as described in Materials and Methods. Samples were subjected to immunoprecipitation without antibody (no ab), with p53 antibody or rabbit IgG (IgG); total lysate was used as a control for PCR amplification (input). **(B, left panel) **p53 binding was tested by using MCP-1 specific primers; **(B, right panel) **p21 primers were used as a positive control. Untreated control cells (-); cells treated with 250 U/ml of TNF-α for 5 h (+).

To investigate whether p53 interacts with its binding site within the MCP-1 enhancer also under *in vivo *conditions, chromatin immunoprecipitation (ChIP) experiments were carried out. For this purpose, we took again advantage of the temperature-sensitive cell system shown in Fig. 2A and 2B. 4Bv cells maintained at 37°C or shifted to 32°C were incubated with TNF-α. After 5 hours, treated and untreated controls were fixed with formaldehyde and DNA sheared samples were immunoprecipitated with antibodies directed against p53. Controls without antibodies or with antibodies against rabbit IgG were included. The origin of the PCR-primers flanking the p53 binding site is outlined in Fig. 5A. As shown in Fig. 5B, MCP-1 chromatin was specifically immunoprecipitated with anti-p53 antibodies only from TNF-α treated cells cultivated at 32°C. In contrast, PCR amplification covering the p53 binding site at the p21 promoter shows a signal after shifting to 32°C, independently of whether TNF-α was added or not. This indicates that, in contrast to p21, p53 binds to the MCP-1 enhancer *in vivo *in a TNF-α dependent manner.

## Discussion

Studying MCP-1 in the context of HPV-induced carcinogenesis, there is apparently a counterselection, which favors the outgrowth of virus-positive cells lacking MCP-1 expression [8,10]. Since high-risk HPVs are known to degrade p53 via the proteasomal pathway [31] and p53 is the most frequently mutated tumor suppressor gene in human cancer [32], we anticipated a potential role of p53 in MCP-1 regulation. This idea was supported by the observation that MCP-1 can only be induced by TNF-α in human keratinocytes immortalized with HPV16 E7, but not in cells carrying E6 alone or together with E7 (Fig. 1A). Hence, co-expression of MCP-1 and HPV16 E6 seems to be mutually exclusive.

The *in silico *predicted p53 binding site within the 5'-regulatory region of MCP-1 (Fig. 1C) was first verified by electro-mobility shift assays (EMSAs), showing complex formation of wild-type but not mutant p53 (Fig. 1D). To further define a p53 dependent mechanism of MCP-1 up-regulation upon cytokine treatment, we used an experimental model in which a temperature sensitive p53val135 variant of p53 can be reactivated from an inactive to an active conformation, simply by shifting the temperature [37]. As shown in Fig. 2A, only in cells where wild-type p53 activity was reconstituted, MCP-1 expression could be induced after incubation with TNF-α. This cannot be attributed to dysfunctional signal transduction or weaker TNFα binding to its cognate receptor after shifting the cells to a lower temperature, since p38 MAP kinase phosphorylation almost followed the same kinetics (Fig. 3B and 3C). Another advantage of this cell system is that p53 reactivation and subsequent p21 induction does not exert a cell cycle arrest (Fig. 3A), most likely due to the presence of very low amounts of retinoblastoma protein (pRB) [37]. Hence, elevated MCP-1 induction was not masked by a potential synchronization effect, which would increase the fraction of cells in G1 of the cell cycle that respond to TNF-α.

To further substantiate a general effect of p53 on MCP-1 regulation, we also used fibroblasts from Li-Fraumeni patients, carrying a germ-line mutation in one p53 allele during early passage, while the remaining wild-type allele of p53 also became mutated after spontaneous immortalization [34,35]. As demonstrated in Fig. 4A, cells cultured in passage 8 still showed a significant MCP-1 induction after TNFα treatment, which was strongly reduced when immortalized cells at passage 160 were examined. A more direct involvement of endogenous p53 in MCP-1 regulation could also be confirmed through direct sh/siRNA knockdown experiments either directly in E7-immortalized keratinocytes (Fig. 1B) or in glioblastoma cells (A172), where in both cases a decreased inducibility of MCP-1 after TNF-α could be observed (Fig. 4D).

However, it should be noted that besides p53, there are still additional transcription factors which may contribute to TNF-α mediated MCP-1 expression in certain cell types. Previous studies have shown that both NFκB and AP-1 are also involved in the transcriptional regulation of MCP-1 [18-21]. DNA binding sites for both have been characterized within the MCP-1 upstream region (Fig. 1C), but AP-1 additionally binds to a stretch of regulatory DNA located downstream of the coding sequence [18]. Here, changes in AP-1 composition and stoichiometry have also profound effects on non-malignant HPV-positive cells. For instance, ectopic expression of c-fos induces tumorigenicity which also resulted in a failure of TNF-α to induce MCP-1 [15]. This indicates that beside p53 and its quantitative reduction in the presence of HPV E6, AP-1 has also to be considered as a major key transcription factor that regulates MCP-1 in non-malignant HPV-positive cells.

Another interrelationship which becomes important in this context is the cross-talk between TNF-α and p53. TNF-α can potentially activate p53 transcription via NFκB binding to its cognate *cis*-regulatory element within the p53 promoter [30]. Moreover, the antiviral activity of TNF-α is based on the induction of interferon-β (IFN-β) [22], which in turn can stimulate p53 both on transcriptional [29] and, as shown recently, also on post-translational levels. The latter is achieved by the induction of the Interferon-Inducible gene IFIXα1, which in turn down-regulates the human homologue of the mouse double-minute gene 2 (mdm2) HDM2, a well-known negative regulator of p53 stability [54]. Such a scenario could explain why in ChIP assays p53 was only found to interact with its binding sites within the MCP-1 regulatory region upon TNF-α treatment (Fig. 5). Transcriptional enhancement of p53 and/or its post-translational stabilization may compensate for lower DNA binding affinity of p53 to the MCP-1 regulatory region as compared to the p21 promoter, whose binding does not require p53 accumulation to the same extent. Consistent with this assumption is the finding that fractalkine, a CX3C chemokine, is also a direct target of p53. Here, there is a dose-dependent chemokine induction, while the steady-state level of p21 is already saturated at lower amounts of p53 [55].

As noted above, there is in fact a functional link between IFN-β and p53 activation, since exogenously added IFN-β even increases p53 levels in HPV18-positive HeLa cells despite E6/E7 expression [29]. The reason why MCP-1 is not induced under these conditions [7-9] is due to the AP-1 dimerization pattern, which exists in a composition typically found when HPV-positive cells undergo malignant progression [9,15]. However, taking advantage of the Li-Fraumeni cell system, TNF-α is strongly inducing IFN-β (see additional file 1), which can explain increased p53 accumulation upon cytokine application in early passage cells (Fig. 4B). Moreover, using non-malignant hybrids made between HeLa cells and human fibroblasts [14], where an AP-1 composition typical for normal cells is restored after somatic cell hybridization [9,15], both IFN-β induction and p53 increase in conjunction with MCP-1 could be discerned (see additional file 1). Tumorigenic segregants derived from the same hybrids in turn lack this property, since MCP-1 induction was as marginal as in late passage Li-Fraumeni cells (Fig. 4A and 4B). Hence, the cross-talk between TNF-α and p53 is apparently mediated by IFN-β, which increases the amount of intracellular p53 to enhance binding to its cognate sequence within the MCP-1 promoter.

In addition, it is also conceivable that transcriptional coactivators may change the local chromatin structure of MCP-1 [56], thereby facilitating p53 binding upon TNF-α treatment. Transcription factors such as NFκB or AP-1 can recruit and interact with coactivators such as CBP/p300, known to possess intrinsic histone acetyltransferase (HAT) activity [57]. CBP/p300 HAT activity is regulated through phosphorylation and is targeted by kinases after TNFα stimulation [58]. CBP/p300 can also interact and acetylate p53, thereby increasing its DNA binding affinity and biological activity [59]. Although previous studies have shown that p53 and NFκB may antagonize each other [60], they were also found to act synergistically in the regulation of gene expression [61]. Hence, the nature of these effects may depend on whether p53 and NFκB compete or cooperate in the recruitment of co-activators such as CBP/p300.

## Conclusions

Our data demonstrate not only a link between p53 and MCP-1 activation, but also suggests that functional p53 inactivation, either via E6-mediated degradation in the context of HPV-induced carcinogenesis [31] or after p53 mutation in other forms of human cancer [32], could affect immunological surveillance by abrogating intercellular communication between somatic cells and cell of the monocyte/macrophage lineage.

## Competing interests

The authors declare that they have no competing interests.

## Authors' contributions

KH performed experiments and wrote parts of the manuscript. BR-O: designed and performed experiments, participated in the discussion, artwork and final manuscript preparation. GB contributed to the ChIP experiments. SYS performed parts of the EMSA experiments. BW designed and supervised the ChIP experiments, contributed to writing of the manuscript. LW designed and supervised EMSA experiments and wrote the corresponding part of the manuscript. FR has designed the study, partially supervised the PhD thesis of KH and wrote the manuscript. All authors read and approved the final version of this manuscript.

## Supplementary Material

Additional file 1 **Expression of interferon-β, in Li Fraumeni fibroblasts and somatic cell hybrids made between HeLa cells and normal human fibroblasts**. **(A)**: RT-PCR of interferon-β (IFN-β) and GAPDH in Li Fraumeni cells (MDAH041) in passage 8 (p:8, p53 mut/wt) and passage 160 (p:160, p53 mut/mut) (see also Fig. 4A). **(B)**: Upper panel: RT-PCR of MCP-1, interferon-β (IFN-β) and GAPDH of non-malignant hybrids (referred as "444") and in derived tumorigenic segregants (referred as "CGL-3"). Lower panel: Western blot. The same filter was consecutively incubated using an anti-p53 (DO-1) and actin antibody. (+) Cells were treated with 250 U/ml of TNF-α for 6 h, (-) untreated control cells. Click here for file
