# Supplementary figures and images for: Regulation of MCP-1 chemokine transcription by p53
Source: Mol Cancer. 2010 Apr 20;9:82. doi: 10.1186/1476-4598-9-82 (PMC2864217; doi:10.1186/1476-4598-9-82)

## Slide 1
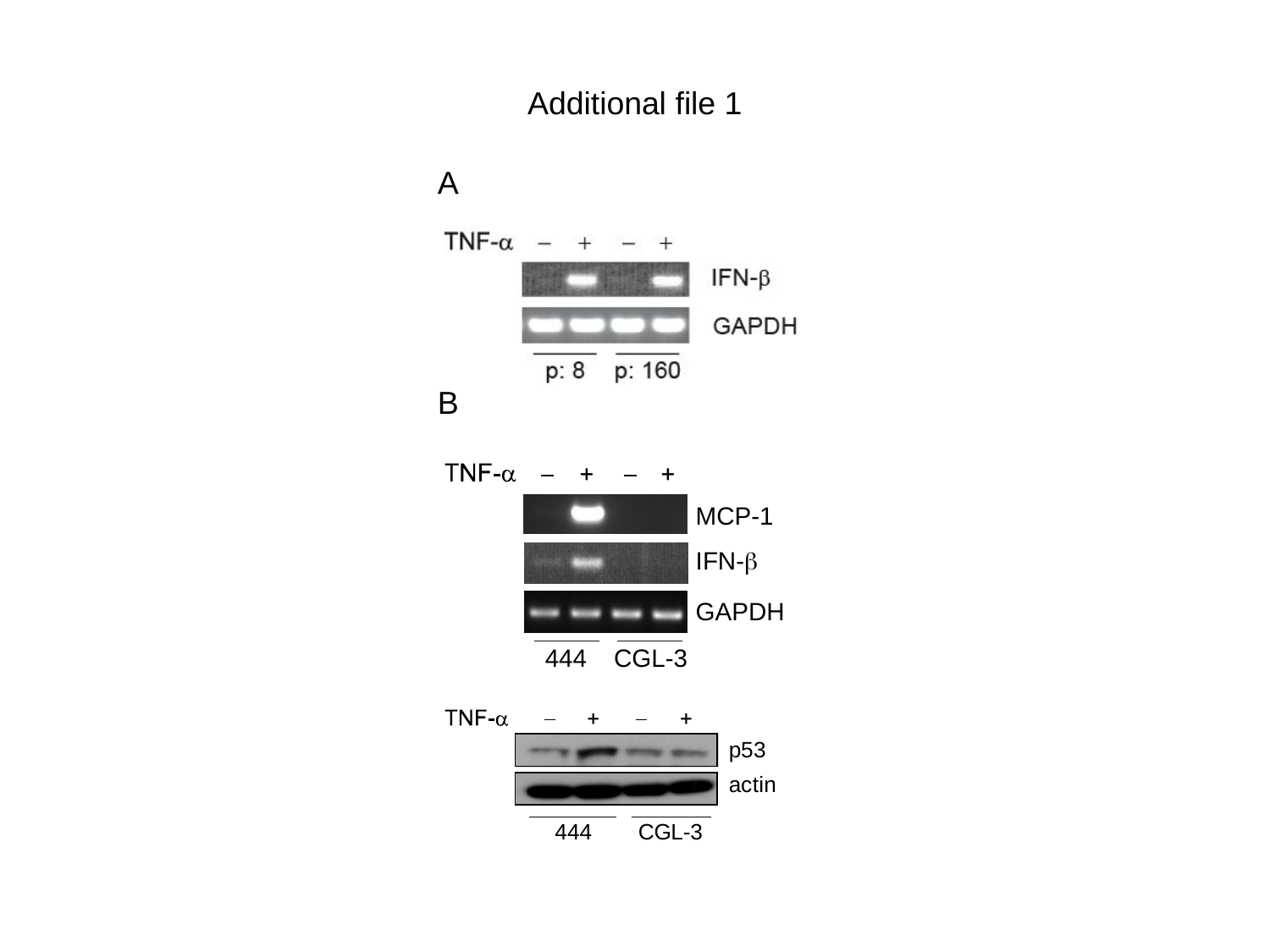

Additional file 1
A
B

Supplement: Additional file 1 — Expression of interferon-β, in Li Fraumeni fibroblasts and somatic cell hybrids made between HeLa cells and normal human fibroblasts. (A): RT-PCR of interferon-β (IFN-β) and GAPDH in Li Fraumeni cells (MDAH041) in passage 8 (p:8, p53 mut/wt) and passage 160 (p:160, p53 mut/mut) (see also Fig. 4A). (B): Upper panel: RT-PCR of MCP-1, interferon-β (IFN-β) and GAPDH of non-malignant hybrids (referred as "444") and in derived tumorigenic segregants (referred as "CGL-3"). Lower panel: Western blot. The same filter was consecutively incubated using an anti-p53 (DO-1) and actin antibody. (+) Cells were treated with 250 U/ml of TNF-α for 6 h, (-) untreated control cells. [file 1476-4598-9-82-S1.PPT]
